# Effect of Manure *vs.* Fertilizer Inputs on Productivity of Forage Crop Models

**DOI:** 10.3390/ijerph8061893

**Published:** 2011-06-01

**Authors:** Giovanni Annicchiarico, Giovanni Caternolo, Emanuela Rossi, Pasquale Martiniello

**Affiliations:** 1 Unit for Extensive Animal Production, Research Council of Agriculture, Via Appia-Bella Scalo, 74085, Muro Lucano, Italy; E-Mails: giovanni.annicchiarico1@entecra.it (G.A.); giovanni.caternolo@entecra.it (G.C.); 2 Centre of Meat Production Breeding, Research Council of Agriculture, Via Salaria 31, 00015, Monterotondo, Italy; E-Mail: emanuela.rossi@entecra.it

**Keywords:** manure, crop models, cropping system, silage and seed production, MFU ha^−1^

## Abstract

Manure produced by livestock activity is a dangerous product capable of causing serious environmental pollution. Agronomic management practices on the use of manure may transform the target from a waste to a resource product. Experiments performed on comparison of manure with standard chemical fertilizers (CF) were studied under a double cropping per year regime (alfalfa, model I; Italian ryegrass-corn, model II; barley-seed sorghum, model III; and horse-bean-silage sorghum, model IV). The total amount of manure applied in the annual forage crops of the model II, III and IV was 158, 140 and 80 m^3^ ha^−1^, respectively. The manure applied to soil by broadcast and injection procedure provides an amount of nitrogen equal to that supplied by CF. The effect of manure applications on animal feeding production and biochemical soil characteristics was related to the models. The weather condition and manures and CF showed small interaction among treatments. The number of MFU ha^−1^ of biomass crop gross product produced in autumn and spring sowing models under manure applications was 11,769, 20,525, 11,342, 21,397 in models I through IV, respectively. The reduction of MFU ha^−1^ under CF ranges from 10.7% to 13.2% those of the manure models. The effect of manure on organic carbon and total nitrogen of topsoil, compared to model I, stressed the parameters as CF whose amount was higher in models II and III than model IV. In term of percentage the organic carbon and total nitrogen of model I and treatment with manure was reduced by about 18.5 and 21.9% in model II and model III and 8.8 and 6.3% in model IV, respectively. Manure management may substitute CF without reducing gross production and sustainability of cropping systems, thus allowing the opportunity to recycle the waste product for animal forage feeding.

## 1. Introduction

Improper use of waste products from animal breeding activity, mainly liquid manure, favours ecological impacts which jeopardize soil and air pollution of environments [1–3]. Utilization of manure in crop growing with appropriate agronomic management practices allows the opportunity to utilize the nitrogen produced by dairy farms for forage crop production [4–7]. Cultivation based on the use of manure in cropping management reduces the input of chemical fertilizers, increases the flow of nutrient cycling farmer and the sequestration of carbon in the Ap horizon [8–10].

Manure appropriately applied to growing crops allows the opportunity to eliminate the nitrogen fertilizer input for crop production [11,12]. Thus, livestock farming based on the use of manure as nutrient reduces the input of [1,9,13,14]. Identifying management practices that will provide a long term agronomic utilization improves the profitably of the manure for agronomic inputs, reducing the impact of the waste products on soil pollution and cost of nutritive feeding values in dairy farms.

The use of manure in dairy farmers of developed countries (Asia, Europe and USA) reduces the dependence on the market for acquiring the feeding product for livestock activity, the cost of farming forage production and the impact of waste products on the environment [6,9,10].

This study evaluated the effect of dairy manure applications for crop nutrient recovery and those of mineral fertilizers on quality of herbage and seed forage production and on biochemical parameters of the topsoil. The experiment aimed to compare, in double yearly forage crops organized in models composed by grasses and legumes species with autumn–winter and spring–summer growing, the effect of an equivalent amount of nitrogen applied with liquid (buffalo and cow) manure with those of inorganic fertilizers on characteristics of herbage and seed forage animal feeding production and and biochemical parameters of topsoil in the Ap horizon.

## 2. Materials and Methods

**Figure 1.**
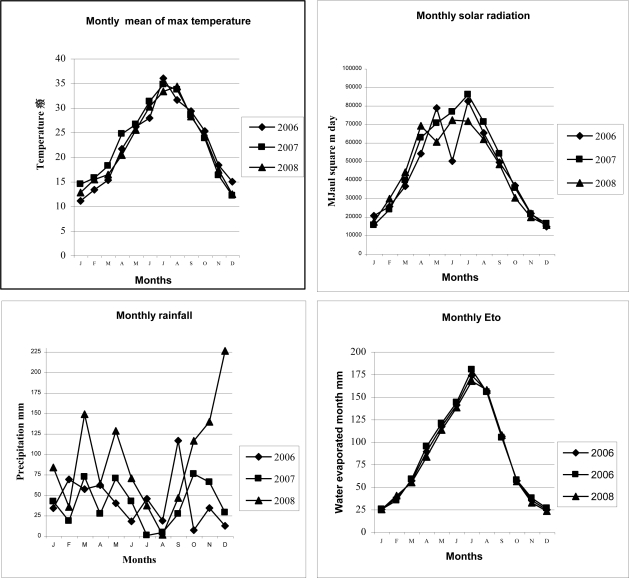
Monthly mean of meteorological characteristics (max temperature, solar radiation, rainfall and evapotanspiration) verified in the years of crops growing.

The field experiment was established between 2006–2008 at the Agricultural Research Council for Animal Production in Monterotondo (E longitude 12°37′ E, Latitude 43°3′ N and 23 m on see level) on a Chromic Vertisol with the following soil characteristics: coarse sand (2–0.2 mm) 310 g kg^−1^; silt (0.02–0.002) 238 g kg^−1^; clay (<0.002 mm) 468 g kg^−1^; pH (water) 8.0; cation exchange capacity 28 cmole g^−1^; total active limestone (CaCO_3_) 83 g kg^−1^; total nitrogen [15] 1.55 g kg^−1^; organic carbon [16] 14.76 g kg^−1^; phosphorous [17] 25 mg kg^−1^; potassium [18] 181 mg kg^−1^. The site where the experiment was established did not have a history of manure application before 2006. The meteorological data for the site where the experiment was carried out was 22.8 °C for annual mean max temperature, 4,650 MJ m^2^ d^−1^ for solar radiation per day, 694 mm of rain per year and 1,018 mm of yearly Eto measured from Class A water pan (Figure 1).

### 2.1. Field Experiment

Straw of the previous wheat crop was removed from the field in the 3rd week of September 2006, before ploughing the topsoil to a depth of 35 cm. After plowing, two manure applications and one fertilizer treatment were applied to the soil.

The manure treatments were applied only on graminaceous crops: barley (*Hordeum vulgare* L.), corn (*Zea mays* L.), Italian ryegrass (*Lolium multiflorum* Lam.) and sorghum (*Sorghum bicolor* (L.) Moench) while legume crops, horse bean (*Vicia faba* L.) and alfalfa (*Medicago sativa* L.), did not receive manure treatments.

The manure used in the experiments was scraped daily and convoyed and stored in a buried metal tank provided with mechanical stirring for blending the liquid mass. Before field spreading, a sample of manure was analysed for determining the content of suspended and dissolved solids (drying at 105 °C for overnight), total nitrogen [19], mineral nitrogen (NH_3_-N) [20], phosphorous and potassium [21]. Organic nitrogen was calculated as difference between total nitrogen and NH_3_-N (Table 1). The volume of manure applied with treatment not considers the content of mineral nitrogen because this fraction will be lost by evaporation before incorporation into the soil.

The total amount of chemical fertilizers (CF) used by dairy farmers for crop cultivation of graminaceous crops are shown in Table 2. The rate of manure application is based on organic nitrogen content (Table 3). The amount of liquid manure for treatment is related to the content of organic nitrogen (Table 1). The m^3^ ha^−1^of manure applied in each application equalled a volume of manure able to supply same amount of inorganic nitrogen of CF treatment (136 kg ha^−1^ for barley and ryegrass, 196 kg ha^−1^ for sorghum and 256 kg ha^−1^ for corn) (Table 2).

The amount of manure (m^3^), equivalent to chemical nitrogen fertilizer, were applied in two treatments: in one run manure application (ORM) the whole amount of nitrogen fertilizer was given at seedbed preparation while in two run manure application (TRM), half the amount was applied at seedbed preparation and a similar amount at the beginning of tiller elongation. Furthermore, for balancing the amount of P_2_O_5_ and K_2_O of the manure treatments to those of chemical application was added 100 kg ha^−1^ of P_2_O_5_ as triple perphosphate and 100 of K_2_O as potassium sulphate. The amount of manure treatment ORM was applied by broadcast before bed preparation while the TRM was supplied by broadcast at bed preparation and the other part at the beginning of tiller elongation spreading out by using a shallow injection depth.

The legume crops (alfalfa and field bean) were fertilised during seed bed preparation with 100 kg ha^−1^ with P_2_O_5_ and K_2_O as triple perphosphate and potassium sulphate, respectively. The fertilizer treatments applied prior seed bed was incorporated in the soil with a field cultivator and tine harrow. Furthermore, the meadow was topdressed with 120 kg ha^−1^ as triple perphosphate in February of 2008.

### 2.2. Cropping System

The field trails are based on three forage crop models with autumn and spring sowing crops and one with winter sowing (meadow of alfalfa) organized in models as reported in Table 3. Model I considers one crop across the duration of experiment while the models II, III and IV consider two growing crops per year. Seed density (kg ha^−1^) and crop systems evaluated in each model are reported in Table 4.

The use of meadow in the cropping system was adopted to compare the effect of manure treatments on soil characteristic and on animal feeding productivity of the models studied. The non utilization of manure treatment on legume forage crops was to avoid the undesirable effect of solid nitrogen on bacterial rhizobia [22] and on establishment of stems [23,24].

The plot basis of autumn and spring sowing in each fertilizer treatment was 15 m long and 20 m wide. The 300 m^2^ plot surface was sown with the graminaceous spp. and alfalfa in rows spaced 12.5 cm wide, 25 cm for horse bean and 50 cm for corn and sorghum. All crops, four times replicated, were planted with a drill in rows. The total number of plots for each fertilizer treatment was 16. The border between plots was 5 m on all sides. The sowing times for autumn and spring crops were the second week of October and June, respectively. Irrigation was based on ETo and applied when evapotranspiration reached 80 mm [25,26]. The amount of water distributed by irrigation system took into account the rainfall and ETo.

Water was applied by a travelling gun with a nozzle pressure of 0.2 MPa and the apparatus was moved by a hydraulic system. In 2007 and 2008 water was applied in four irrigations of 100 mm each. In each plot, to avoid any border effects, samples for biomass, grains and soil characteristic determinations were harvested at the middle of the plot.

The alfalfa forage meadow was harvested when more than 80% of tillers in the plot had flowered while the I harvest in barley and Italian ryegrass, was at heading (2nd week of April) and horse bean at the beginning of flowering (2nd week of May). In all crops, prior to harvest, mean plant height (cm) was a determined by measuring at random six main tiller values taken from ground level to the top of plant.

The II harvest in annul autumn sowing crops, was made 30 days later. Forage dry matter (DM, g m^−2^) and crop growth rate of dry matter accumulation per day (CGR, g m^−2^ d^−1^) from seed emergence to harvest, was assessed on fresh herbage from each experimental plot of the treatments. In each harvest, the herbage of 1 m^2^ surface was manually mowed from ground level and weighed. The moisture (%) content and leaf stem proportion at harvest were determined from a fresh forage sample, approximately of about 500 g and 50 stems, respectively, taken at random from the biomass. The sample for moisture determination at harvest was dried at 65 °C with forced ventilation until the biomass weight remained constant while the leaf-stem ratio (L/S) was determined from the weight of 50 stem leaves and whole stems expressed as a percentage.

The crops of barley, horse bean and sorghum used for seed consumption were harvested at seed physiological maturity of the seeds in the fructiferous organ. The traits determined on plot basis were: seed yield (t ha^−1^ at 13 g kg^−1^ standard moisture content) and 1,000 seed weight while seed yield components (stems m^−2^; seeds spike^−1^; and harvest index, ratio of seed yield and biomass weight express in percentage) were determined on samples of tillers harvested from 1 m^2^ sections before threshing the plot.

From each treatment plot, a sample of about 200 g of dry matter and seed harvest was ground (PBI Tetator Cyclotec 1,093 sample mill, Made in Sweden) with a mesh screen of 1 mm Ø for providing flour for laboratory analyses. The samples were stored in cell room at 4 °C until qualitative laboratory determinations.

The nutritive values, expressed in milk forge unit (MFU), were evaluated chemically. The forage qualitative parameter determined were: crude protein (CF) [15], neutral-detergent fibre (NDF), acid-detergent fibre (NDF) and acid-detergent lignin (ADL) [27]. The herbage MFU was assessed according to the method of Andrieu and Weiss [28].

### 2.3. Soil Sample for Biochemical Determinations

Soil samples from each treatment were collected at the beginning and at the end of experiments (October of 2006 and 2008, respectively). A soil core samples of 0–35 cm of the Ap horizon with a 60 mm diameter, was picked up with mechanical equipment for physicochemical determinations. Each sample was made mixing four cores soil randomly drilled from the plot surface. After thorough manual root separation, the samples were air-dried, sieved with a mesh screen of 2 mm of Ø and stored at 4 °C in a cool room until they were used for laboratory determinations [29]. The total number of harvests was 64 at the beginning and as many as at the end of the experiment. The physical soil determinations were determined according to Day [30] hydrometer methodology while those of soil chemical parameters were assed according to the methodology of Kjeldahl [15], total nitrogen; Walkley and Black [16], organic carbon; UNICHIM [18], potassium; Olsen *et al.* [17], phosphorus; Druineau [31] and Géhu and Franck [32], cation exchange capacity and pH on liquid extract of 1:2.5 soil/water solution.

### 2.4. Statistical Analysis

Data of traits produced by models, fertilizer treatment, winter and spring sowing in the two years were analysed adopting 2 × 3 × 2 × 4 factorial design (two years; three fertilization treatments; two sowing date and four crop models) laid out in a split-split-split model with four randomized complete blocks [33]. The main plot was fertilizer treatment with sowing date in the subplots. Forage crop models were randomised in the subsubplots. The ANOVA used a mixed model, with years and replications as random effects and fertilizer, sowing date and model as fixed effects. Because the interaction effects of year with other main factors were not significant, the data presented in the results, tables and figures are shown as a mean of the years. Mean of fertilizers models their two- or three-factor interactions were separated using Fisher’s protected last significant difference (LSD) at P ≥ 0.05 level of probability while the effect of fertilizer treatments within models and those of harvests in autumn sowing crops, were tested with Duncan’s Multiple Range Tests at P ≥ 0.05 probability level [33].

## 3. Results

Figure 1 provides an overview of the prevailing weather conditions during the growing periods of the experiments. The environmental factors reached favourable condition for plant growth during Spring and were gradually unfavourable in the Spring-Summer months (weather conditions become gradually hot and dry in Summer). Among weather events, rainfall was the more variable parameter across the month than temperature, Eto and solar radiation (Figure 1).

The effect of experimental treatments differently influenced the development of barley and Italian ryegrass crops while in annual and perennial legume, where the manure applications were not applied, the statistical significance among traits was lacking (Tables 5 and 6).

In winter sowing crop model II and model III, the traits biomass, moisture at harvest, L/S and MFU in the first and second harvest evidenced significant effect among ORM, TRM and CF treatments (Table 5).

Particularly in barley, the lower moisture content in the II harvest in the plant treated with CF evidenced short vegetative cycle due to lower levels of nutrient availability in the soil which reduced the physiological process in plant development in comparison to those of manures.

The higher number of significant traits in barley and Italian ryegrass observed in I harvest rather than the II one was ascribed to the higher effect of manure and those of fertilizer on early phenological stage of plant development. In barley at I harvest, the CF increased biomass yield by 3.2% in ORM and 2.7% in TRM applications while opposite trend of development was observed in the II harvest (6.6% in ORM and 3.3% in TRM lower than CF). In Italian ryegrass under I harvest, the effect of manure on plant was evident only in TRM which increased biomass by 3.6% over ORM and 1.6% over CF while in the II harvest, the effect of ORM was 15.9% and 8.8% higher than TRM and CF, respectively (Table 5).

The moisture at harvest in CF, in the first harvest, was 3.1% in barley and 4.1% in Italian ryegrass higher than the mean of manure treatments while in the II harvest the trait was reduced in barley by 7.5% and increased by 2.3% in Italian ryegrass.

The physiological process of plant development under manure treatments in comparison to CF had longer metabolic activity. Particularly in barley, the lower moisture content in the II harvest, in the plant treated with CF, evidenced short vegetative cycle due to lower source of nutrient availability in the soil which reduced the physiological process of plant development in comparison to those of manures.

The manure application: ORM in barley, in the I harvest favoured the development of L/S ratio in comparison to those of CF (ORM 4.7% higher than those of CF); an opposite trend was observed in the Italian ryegrass harvest (CF was 5.1% than ORM and TRM,) (Table 5). Different behaviours of L/S ratio was found in barley at I harvest in which the effect of CF increased by 17.5% the value of ORM and 3.8% those of TRM while in the II harvest the value of trait was reduced by 9.1% the mean of manure applications.

The effect of crop gross product biomass and seed from the models was differently affected by manures and CF treatments. The MFU content in biomass of in winter sowing crops was more related to plant development than manures and CF applications (MFU of II harvest was higher than I harvest 3.4% in barley and 5.0% in Italian ryegrass) (Table 5). Similar trend in MFU content among fertilizer treatment was observed in spring sowing crops (Tale 6). The content of MFU in seeds of autumn sown crops was positively influenced by manures and those of spring sowing by CF (Table 7). The effect of manure and CF applications played a different physiological role on dry matter crop growth rate (CGR) between the crops. The CGR in I harvest of CF treatment in barley and in Italian ryegrass was lower than those of ORM and TRM applications (10.8 % and 7.0% in barley and 16.0% and 23.8% in Italian ryegrass, respectively). By contrast, the gap among manures and CF in the II harvest was reduced (5.0% and 6.2% lower than manures in barley and ryegrass, respectively).

In agreement with the other results, the variation between CGR values of in the I and II harvests was a consequence of soil nitrogen availability to the crops in topsoil [34–38]. Thus, as seen in canola (*Brassica napus* L.) by Sharif *et al.* [38], the manure treatments increased the nitrogen availability in the soil rather than CF, conferring to the plant higher physiological activity and consequently higher CGR.

The lack of statistical significance in all the traits of alfalfa meadow across the harvests in the plots of ORM, TRM and CF treatments evidenced a uniformity of the soil where the experiment was established (Table 6).

**Figure 2.**
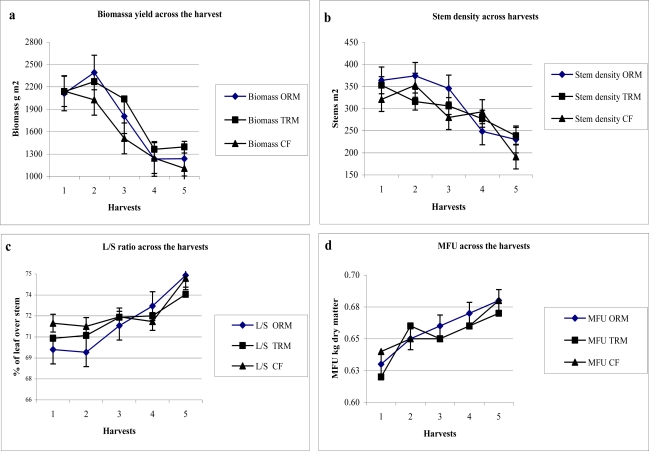
Behaviour of MFU, L/S ratio, stem density and biomass yield traits in alfalfa across the harvest during the period of evaluation.

The yearly alfalfa mean of biomass production derived from the sum of the five biomass harvests applied during the period of plant development was lower than those of other crops (Table 6). The biomass yield of second harvest was 4.4%, 20%, 42.1% and 44.1% higher, respectively than first, third, fourth and five harvest (Figure 2a) while the stems m^−2^ in the fist two harvests showed small variation (347 stems m^−2^) in comparison to the following third, fourth and fifth harvest in which the value of trait was reduced by 10.5% 12.2% and 29.2%, respectively (Figure 2b). The amount of L/S ratio (Figure 2c) and MFU (Figure 2d) increased linearly through the harvest passing from 70.3% and 0.63 in first harvest to 74% and 0.68 in the fifth, respectively. The moisture content of biomass at harvests was about 72%. The variation observed across the harvest was ascribed to limitation of water supply, senescence, light competition, efficiency of physiological process and persistency of plant during growing season [39,40].

The ORM, TRM and CF influenced seed production of barley and sorghum crops while horse bean, because it was not treated with manure, the trait was not significant (Table 7). In barley, the CF increased seed yield by 25.6% over TMR and 32.5% over ORM while in sorghum seed the trait was reduced by 2.1% under OMR and by 7.0% under TMR (Table 7).

The reduced effect of manure on seed production applications on winter sowing crops in comparison to those of spring sowing was ascribed to the constrain of unfavourable weather condition on the mineralization of manure organic compounds which weakened the availability of nitrogen uptake for barley rather than for sorghum crop development (Figure 1).

The effect of CF was expressed better than that of manures (mean of ORM and TRM), in barley seed yield component traits (5.7%, stem m^−2^; 5.1%, 1000 seed weight; 3.1%, seed spike; and 25.5%, harvest index) while the opposite trend was observed in seed yield components of sorghum seed. The favourable effect of manures over CF application in sorghum seed yield was promoted by mineralization of the manure’s organic compounds and consequently large availability of removal nitrogen for development of aerial biomass and seed yield components (23.4%, stem m^−2^; 1.5%, seed per head; and 11.6% harvest index) (Table 7). The effect of ORM, TRM and CF on silage corn and sorghum crops had little affect on L/S, while in sorghum seed used for silage consumption the effect of ORM and TRM influenced leaf development rather than CF treatments [11] (Table 6).

Comparing autumn sowing to the spring crops, as effect of environmental conditions occurred during the vegetative growth (Figure 1), the mean of MFU ha^−1^ over crops of OMR, TMR and CF treatments was lower in silage (29.1%, 34.0% and 29.9%, respectively) and in seed yield (35.6%, 25.3% and 11.4%, respectively) than spring sowing crops (Table 8). The mean among fertilizer treatments of MFU ha^−1^ produced by silage in autumn sowing, was in horse bean (model IV) higher than 37.5%, Italian ryegrass (model II) and 42.3% and in barley (model III) while the MFU ha^−1^ of horse bean (model IV) produced by seed was 22.8% higher than barley (model III) (Table 8). Furthermore in barley, the MUF ha^−1^ derived from seed under CF treatment was 5.2% higher and 5.2% lower than ORM and TRM, respectively. As expected, the MFU ha^−1^ produced by alfalfa (model I) and by horse bean (model IV) was not significant among plots.

The effect of CF treatment on MFU ha^−1^ production in sorghum seed for silage consumption was lower (47.4% and 55.0%) than silage sorghum and corn, respectively. The mean of MFU ha^−1^ from sorghum seed as seed consumption was 7.6% higher than those of silage utilization. The TMR application positively affected the yielding traits in sorghum seed (3.5% and 6.1% in ORM and 4.1 and 6.1 in CF for silage and seed consumption, respectively) (Table 8).

**Figure 3.**
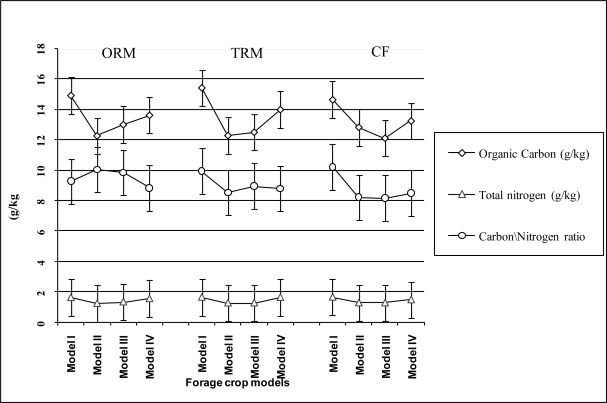
Biochemical trait values across the models at end of experiments in topsoil of the manures and CF application treatments.

The mean of trait organic carbon and total nitrogen (expressed in g kg^−1^) and C/N (pure number) were, 15.0, 1.63 and 9.8 under alfalfa and 13.4, 23.2, 1.58 and 9.0 under horse bean, respectively, while under grass crops these values were strongly stressed (Figure 3). The residual gap existing in biochemical traits among models was ascribed to mineralization of manure compounds brought by manures in the topsoil of the crop system. The increase of the biochemical values of parameters present at the beginning of experiments in alfalfa (model I) (see Materials and Methods section) was due to a rhizosphere microbial activity while their decrease in horse bean (model IV) was a consequences of mineralization process of organic matter for provide nutrient needed for plant development of spring sowing sorghum for silage production (model IV). Almost similar soil content found in organic carbon and total nitrogen in the model II and model III in manures *vs.* CF application (12.3% *vs.* 12.5% in organic carbon and 12.6% *vs.* 12.6% in total nitrogen) was a consequence of the mineralization process of the organic components present in topsoil and those applied with manure treatments for satisfying the demand of nutrients required for development of autumn and spring sowing crops. The effect of horse bean (model IV) increased, in the spring sowing silage sorghum model under manures in comparison to CF applications, the content of organic carbon by 4.4% and 11.3% the total nitrogen (Figure 3). The residual content of organic carbon and total nitrogen in model II was reduced by 16.6%, and 20.5% under manure (mean over treatments) and 12.0%, and 18.7% under CF the values present in the model I, respectively.

The trend of variation in the biochemical traits in model III was quite similar to those observed in model II. The residual effect of biochemical compounds in topsoil in the model IV, as effect of annual crop legume on rhizosphere, resulted less stressed than model II and model III (mean of organic carbon and total nitrogen over CF application was lower 7.8%, 7.9% than model IV, respectively). The higher values of the C/N ratio observed under models carried out under manure application (9.6, 8.7, and 8.3 in ORM, TRM and CF, respectively) evidenced a higher microbial activity under manure applications which release large quantities of nutrients uptake advantaging the development of crops [41,42].

## 4. Discussion

The results evidenced an agronomic opportunity to use dairy farm manure for forage feeding production reducing the ecological damage caused by the waste product. The nitrogen uptake from manure treatments (mean of ORM and TRM) achieved an increase of biomass in graminaceous crop production by 5.9%, in comparison to CF (Tables 5 and 6). Sutton *et al.* [11] in corn, Cherney *et al.* [43] in orchardgrass (*Dactylis glomerata* L.) and tall fescue (*Festuca arundinaceae* Shreb.) and Blanchet and Schmitt [44] in alfalfa, corn, sorghum and soybeans [*Glicine max* L. (Merr.)] observed the different behaviour of manures treatments on crop, in comparison to those of CF, that was ascribed to the influence of temperature and soil moisture on the manure mineralization process which effect nitrogen removal for aerial plant growing. The variation between CGR values across phenological stage of plant maturity in the I and II harvests, according to the results of Greenwood *et al.* [34], Sharif *et al.* [38] in canola (*Brassica napus* L.) and Shukla *et al.* [45] in Indian mustard (*Bassica juncea* L.), Oscar and Tollenar [36] and Amanullah *et al.* [37] in corn, was ascribed to senescence of leaves and to the expiring of nitrogen source in the soil which reduced the availability of nitrogen uptake to the crop (Table 5).

The beneficial effect of grasses crops due to manure application rather than CF, according to the results found by Bittman *et al.* [41], Cherney *et al.* [43] and Zhang [10], was allowed by mineralization process of organic components brought in the soil by manure applications which provide elements and nutrients for better plant development. According to the results of the authors, can be assumed that the amount of liquid manure applied in the experiments was able to significantly sustain the biomass of winter and spring sowing graminaceous crops used in the models better than CF application (manures increased the biomass yield of barley, Italian ryegrass, corn and sorghum by 5.1%, 2.9%, 9.9% and 17.9%, respectively, compared to CF) (Tables 5 and 6). The nitrogen benefit acquired from manure utilization in autumn and spring sowing of graminaceous spp. in comparison to CF, was higher for forage than seed production (biomass yield of manures was higher than those of CF 5.0%, 1.2%, 2.2%, 3.8% and 12.1% in barley, Italian ryegrass, corn, sorghum seed for silage consumption and silage sorghum, respectively) (Tables 5 and 6).

In agreement to the results obtained by Zhang *et al.* [10] in bromegrass (*Bromus inermis* Leyss.) and oat (*Avena sativa* L.), the contrasting effect between inorganic fertilizers (CF) and liquid manure (ORM and TRM) observed in seed yield production in barley and sorghum seed (29.1% and 4.6% higher and lower than mean of manure treatments, respectively), may be due to lack of beneficial effect related to cool temperatures occurred during the growing and development of the two crops (Figure 1). The effect of weather factors, mainly temperature and humidity, during barley and sorghum development limited the mineralization of nutrient released by manure which supply lower amount of removal nitrogen availability in barley than seed sorghum (Figure 1, Table 7).

Considering the gap between dry matter production in autumn sowing crops of manure applications (mean of ORM and TRM) in the II harvest [barley (1,251 g m^−2^), Italian ryegrass (1,326 g m^−2^)] with those of spring sowing [corn (3,067 g m^−2^), seed sorghum for silage consumption (1,264 g m^−2^) and silage sorghum (3,043 g m^−2^)], it is possible to assume that the mineralization process of the manure and availability of cycling products in soil was related to environmental factors rather than the amount of biological product buried by manure in the soil (Tables 5 and 6). Furthermore, the higher dry matter production seen in spring than in autumn sowing and the higher TRM application than ORM (1.6% in corn, 4.6% in seed and 13.4% in silage sorghum) evidenced that the amount of nutrient available to the crops, under favorable environmental condition, are related to the available manure present in topsoil (Tables 5 and 6). The results of the experiments evidenced that the benefits of livestock liquid manure on crop yield and qualitative characteristics of graminaceous grasses were similar to those reported by other studies [10,12,46–49].

In agreement with Boote *et al.* [50], Kagata *et al.* [51] and Søresen *et al.* [52] because the manures interfere differently with plant development than CF applications, the MFU and MFU ha^−1^ of crop gross product harvested from the models was differently affected by manures and CF treatments (Tables 5, 6 and 7).

The higher mean over models of MFU ha^−1^ from silage under manures rather than CF (mean of ORM and TRM higher 9.2% in winter and 11.4% spring sowing crops than CF) was related to the environmental weather conditions on the crop growth of the model (Table 8). These results were in line with those reported by Blanchet and Schmitt. [44] and Russelle *et al.* [12] in corn and sorghum experiments grown under manure broadcast before sowing and whorl of the plant development. According to the authors, the higher mean of biomass production under manures than CF applications (biomass mean values of ORM and TRM 9.2% in corn and 2.1% in sorghum seed for silage, 7.3% sorghum seed and 26.7% on sorghum silage higher than CF applications) was achieved by a mineralization process which provides nitrogen removal and nutrient compound for plant development whose biomass production exceeded those of CF supply (Table 6).

The discrepancy observed in the crops with autumn and spring sowing was a consequence of weather condition that occurred during the crop development. The lower effect of manure applications in autumn rather than spring sowing, as evidenced by Cherney *et al.* [43] in perennial grasses in autumn sowing crops and Russelle *et al.* [12] in corn are linked to the environmental factors involved in mineralization processes (temperature and soil moisture) of soluble solid present in ORM and TRM applications.

The advantages in using manure applications increased the nutrient uptake favouring crop biomass and MFU ha^−1^ production and reduced the organic carbon and total nitrogen in the soil in the model II and model III in comparison to model I and model IV (Figure 3). The growing crops of models II and III in comparison to model I and IV stressed the content (mean over models) of organic carbon and total nitrogen in topsoil by 13.3% and 20.6% in ORM and TRM and 11.9% and 21.3% in CF treatment.

According to the results reported by Bittman *et al.* [41] and Min *et al.* [42], the higher benefit evidenced in the model II under ORM and TRM applications than those of CF, was ascribed to advantages in uptake large quantity of nutrients and to greater response of crops to manure rather than other models.

The combination of manure management with agronomic crop rotations may avoid the constraining effects of long term manure application reported by Min *et al.* [53] and Newton *et al.* [4] on soil and biomass quality. The authors, in continuous intensive manure applications on double graminaceous multi-cropping systems (bermudagrass, *Cynodon dactylon* L.) and corn and rye (*Secage cerale* L.), determined detrimental effects on selection of soil biological and chemical properties of biomass which reduced soil quality and enhanced carbon sequestration in top soil.

According to Newton *et al.* [4], Edmeades [54] and Yolcu *et al.* [49] the rotation among intensive forage cropping systems based on perennial legumes (model I), double graminaceous crops (model II and model III) and legume and graminaceous crops (model IV) in dairy livestock intensive cropping systems protected the soil quality and avoids selection among microbes preserving the biodiversity in the soil rhizosphere. Based upon the results of this study double annual applications of manure can be made upon the annual forage crops species evaluated in the experiments at rate of 158 m^3^ ha^−1^ in model II, 140 m^3^ ha^−1^ in model III and 80 m^3^ ha^−1^ in model IV without effects the quality of herbage production and quality and content of biochemical characteristics in topsoil. The use of manure with appropriate practices of managements for forage crops production in multi-cropping system in dairy farms, favoured the exploitation for agronomic purpose the manure produced from husbandry activity saving the microbial biodiversity in topsoil and may represent an effective source of plant nutrients for recycled forage production, in dairy farm husbandry.

## 5. Conclusions

Dairy farmer liquid manure may substitute for CF applications without interfering with the feeding quality of crop gross product and biochemical parameters of topsoil. The beneficial effect of ORM and TRM applications on winter sowing crops, in comparison to CF treatment, favoured biomass production of barley and Italian ryegrass in both harvests. The ORM application advantages the plant development of models used for forage while TRM those of seed consumption.

The lower MFU ha^−1^ of winter sown crops in comparison to spring ones was due to a favourable weather condition (mainly temperature) which favoured mineralization of manure in the soil achieving large nutrient availability for crop development.

The combination of appropriate agronomic management practices of manure with grass-legume crop systems, avoids the negative effect of long term manure on soil and herbage quality, reduces the dependence of nitrogen fertilizer, and acquisition of products (hay, legume and grass seeds) for animal feeding by the market and refrains the selection of the biological activity process (maintaining the biodiversity) among rhizobial bacteria in the soil. The management of manure with ORM and TRM applications on the crop used in the models, in comparison to CF, favours sustainable cultivation of dairy production, reduces the stringent quality of environment protection requirements, minimizes the negative impacts of manure on soil pollution and changes the consoderation of manure from a waste to a resource product.

## Figures and Tables

**Table 1.** Physicochemical characteristics of manure fertilizer applied in the experiments.

	**Total solid suspended kg m^−3^**	**Total solid dissolved kg m^−3^**	**Organic Nitrogen kg m^−3^**	**Phosphorous kg m^−3^**	**Potassium g m^−3^**
Mean	6.20	1.34	2.53	0.17	0.39
Sx*	19	0.6	0.19	0.02	0.09

* Standard error.

**Table 2.** Inorganic fertilized applied to the crops in the conventional standard management.

		**Fertilized applied to crops**				
		**Before sowing**			**Topdressing**	

**Crop**	**Model**	**Nitrogen kg ha^−1^**	**Phosphorous kg ha^−1^**	**Potassium kg ha^−1^**	**Nitrogen kg ha^−1^**	**Phoshorous kg ha^−1^**
	Autumn-winter crops					
	
Italian ryegrass-Barley	II–III	36 as Ammonium phosphate	96 as Ammonium phosphate	100 as Potassium sulphate	100 as Ammonium nitrate	
	
Horse bean	IV		100 as Triple per phosphate	100 as Potassium sulphate		

	Spring-summer crops					

Alfalfa	I		92 as Triple per phosphate	100 as Potassium sulphate		120 as Triple per phosphate
	
Corn	II	36 as Ammonium phosphate	96 as Ammonium phosphate		220 as urea	
	
Sorghum	III–IV	36 as Ammonium phosphate	96 as Ammonium phosphate		160 as urea	

**Table 3.** Amount of compounds applied in autumn-winter and spring-summer crops with ORM at seedbed and TRM at seedbed and stem elongation manure treatments.

**Crop**	**Model**	**Manure Applied m^3^ha^−1^**	**Solid body kg ha^−1^**	**Nitrogen kg ha^−1^**	**Phosphorous kg ha^−1^**	**Potassium kg ha^−1^**
				ORM		

	Autumn-winter crops					
	
Italian ryegrass-Barley	II–III	60	453	150	10.3	0.02

	Spring-summer crops					
	
Corn	II	98	739	250	16.9	0.04
	
Sorghum	III–IV	80	603	200	13.8	0.03

				TRM		

	Autumn-winter crops					
	
Italian ryegrass-Barley	II–III	30	222	75	5.2	0.01
		30	226	75	5.2	0.02

	
	Spring-summer crops					
	
Corn	II	49	370	125	8.4	0.02
		49	370	125	8.4	0.02
	
Sorghum	III–IV	40	302	100	6.9	0.02
		40	302	100	6.9	0.02

**Table 4.** Seed sowing density of autumn-winter and spring-summer forage crops models adopted in the experiments.

	**Forage crops belong to the model**			

**Model**	**I**	**II**	**III**	**IV**
	Autumn-winter crops			

Crop	----	Italian ryegrass	Barley	Horse bean
Seed sown (geminated seed m^−2^)		750	400	80

	Spring-summer crops			

Crop (geminated seed m^−2^)	Alfalfa	Silage corn	Seed sorghum	Silage sorghum
	750	12	25	50

**Table 5.** Autumn sowing crops biomass characteristics of the manures *vs.* inorganic fertilizer treatments across the harvests.

**Crop Traits**	**I harvest**			**II harvest**		
	**ORM**	**TRM**	**CF**	**ORM**	**TRM**	**CF**
Barley						

Plant height (cm)	118 a	111 b	109 b	118 a	115 a	121 a
Stem m^−2^ (n)	440 a	416 b	396 b	349 a	329 a	354 a
Herbage biomass (g m^−2^)	5,014 b	5,038 b	5,178 a	3,223 a	3,113 a	3,009 b
Moisture (%)	78 b	79 b	81 a	60 a	61 a	56 b
L/S (%)	49 a	47 b	47 b	38 b	38 b	40 a
MFU (kg DM)	0.77 a	0.76 b	0.78 a	0.80 a	0.79 b	0.80 a

Italian ryegrass						

Plant height (cm)	103 a	109 a	104 a	138 a	133 a	128 a
Stem m^−2^ (n)	556 a	524 a	433 b	399 a	395 a	369 b
Herbage biomass (g m^−2^)	5,232 c	5,425 a	5,336 b	3,891 a	3,274 c	3,550 b
Moisture (%)	83 b	82 b	86 a	67 a	62 b	66 a
L/S (%)	43 c	50 b	52 a	39 a	38 a	35 b
MFU (kg DM)	0.70 b	0.71 a	0.71 a	0.75 a	0.74 b	0.74 b

Horse bean *						

Plant height (cm)	135 a	132 a	132 a	144 a	140 a	143 a
Stem m ^2^ (n)	59 a	55 a	49 a	45 a	46 a	45 a
Herbage biomass (g m^−2^)	5,268 a	4,894 a	5,255 a	5,973 a	5,999 a	5,729 a
Moisture (%)	86 a	86 a	86 a	81 a	81 a	80 a
L/S (%)	36 a	34 a	33 a	41 a	42 a	41 a
MFU (kg DM)	0.76 a	0.75 b	0.76 a	0.78b	0.79 a	0.79 a

* Value of main plot in experimental design without manure application; Mean among fertilizer treatments with the same letter are not statistical significant at Duncan’s Multiple-Range Test at P = 0.05 level of probability.

**Table 6.** Spring sowing crops biomass characteristics in the ORM, TRM and CF treatments.

**Bioagronomic traits**	**Experimental treatments**			**LSD 0.05**
	**ORM**	**TRM**	**CF**	

	**Corn**			
Plant height (cm)	256	256	245	9
Stem m^−2^ (n)	7.8	8.3	8. 2	ns
Herbage biomass (g m^−2^)	7,268	7,515	6,756	457
Moisture (%)	58	59	59	ns
L/S (%)	32	31	31	ns
MFU (kg DM)	0.88	0.87	0.89	0.1

	Seed sorghum for silage consumption			

Plant height (cm)	107	105	96	9
Stem m^−2^ (n)	21	20	19	ns
Herbage biomass (g m^−2^)	2,767	2,946	2,788	149
Moisture (%)	56	57	57	ns
L/S (%)	40	39	32	3.7
MFU (kg DM)	0.85	0.84	0.83	0.1

	Silage Sorghum			

Plant height (cm)	265	266	253	13
Stem m^−2^ (n)	36	33	33	ns
Herbage biomass (g m^−2^)	6,483	7,186	6,010	933
Moisture (%)	56	55	57	ns
L/S (%)	20	19	21	ns
MFU (kg DM)	0.69	0.68	0.67	0.1

	Alfalfa *			

Plant height (cm)	73	72	72	ns
Stem m^−2^ (n)	278	279	276	ns
Herbage biomass (g m^−2^)	1,755	1,839	1,785	ns
Moisture (%)	68	70	69	ns
L/S (%)	71	72	71	ns
MFU (kg DM)	0.66	0.65	0.66	ns

* Value of main plot in experimental design without manure application; ns = statistically not significant

**Table 7.** Autumn and spring crop sowing seed yield and seed yield components in the ORM, TRM and CF treatments.

**Bioagronomic traits**	**Experimental treatments**			**LSD 0.05**
	**ORM**	**TRM**	**CF**	

	**Barley**			
Seed yield (g m^−2^)	459	506	680	98
Stem m^−2^ (n)	405	370	411	12
1000 seed weight (g)	36	38	39	3
Seeds spike (n.)	31	31	32	2
Harvest Index (%)	18	22	27	4
MFU (kg DM)	1.01	1.02	1.00	1

	Sorghum			

Seed yield (g m^−2^)	595	627	583	18
Stem m^−2^ (n)	24	23	18	2
1000 seed weight (g)	11	10	10	ns
Seeds spike (n.)	723	730	716	15
Harvest Index (%)	23	29	23	3
MFU (kg DM)	1.01	1.02	1.03	1

	Horse bean *			

Seed yield (g m^−2^)	351	364	360	ns
Stem m^−2^ (n)	46	51	46	ns
1000 seed weight (g)	307	308	318	ns
Seeds spike (n.)	3.1	2.9	2.9	ns
Harvest Index (%)	14	15	12	ns
MFU (kg DM)	1.07	1.05	1.07	ns

* Value of main plot in experimental design without manure application; ns = statistically not significant.

**Table 8.** Mean crop MFU ha^−1^ production across the autumn and spring models in the manure fertilizer treatments.

**Crop utilization Model**	**OMR**			
	**I**	**II**	**III**	**IV**

	Autumn-winter crops			
Silage		6,189 b	6,117 b	10,002 a
Seed			3,650 a	5,089 b
LSD 0.05			350	478
	Spring-summer crops			
Silage	11,583 b	13,993 a	5,613 d	10,735 c
Seed			6,008	
LSD 0.05			580	
Total MFU ha^−1^				
Silage	11,583 d	20,182 a	11,730 b	20,737 c
Seed			9,658 a	5,089 b
LSD 0.05			670	897

**Crop utilization Model**	**TRM**			
	**I**	**II**	**III**	**IV**

	Autumn-winter crops			
Silage		6,818 b	5,138 c	9,840 a
Seed			4,398 b	5,161 a
LSD 0.05			654	769
	Spring-summer crops			
Silage	11,954 b	14,050 a	5,815 c	12,217 b
Seed			6,398	
LSD 0.05			754	
Total MFU ha^−1^				
Silage	11,954 d	20,868 a	10,953 b	22,057 c
Seed			10,796 a	5,161 b
LSD 0.05			896	986

**Crop utilization Model**	**CF**			
	**I**	**II**	**III**	**IV**

	Autumn-winter crops			
Silage		5,191 b	5,551 b	9,288 a
Seed			3,850 b	5,800 a
LSD 0.05			532 b	785

**Crop utilization Model**	**CF**			
	**I**	**II**	**III**	**IV**

	Spring-summer crops			
Silage	10,461 b	12,630 a	5,581 d	9,411 c
Seed			6,007	
LSD 0.05			389	
Total MFU ha^−1^				
Silage	10,461 d	17,821 a	10,132 b	18,699 c
Seed			9,857 a	5,800 b
LSD 0.05			679	897

Means with the same small letter among models are not statistical; significant at Duncan’s Multiple Range Test at P ≥ 0.05 probability level.
